# The balance of VEGF-C and VEGFR-3 mRNA is a predictor of lymph node metastasis in non-small cell lung cancer

**DOI:** 10.1038/sj.bjc.6603209

**Published:** 2006-06-06

**Authors:** H Takizawa, K Kondo, H Fujino, K Kenzaki, T Miyoshi, S Sakiyama, A Tangoku

**Affiliations:** 1 Department of Oncological and Regenerative Surgery, University of Tokushima School of Medicine; 3-18-15 Kuramotocho, Tokushima 770-8503, Japan

**Keywords:** lung neoplasms, vascular endothelial growth factor-C, vascular endothelial growth factor receptor-3, lymph node metastasis

## Abstract

A positive association between vascular endothelial growth factor-C (VEGF-C) expression and lymph node metastasis has been reported in several cancers. However, the relationship of VEGF-C and lymph node metastasis in some cancers, including non-small cell lung cancer (NSCLC), is controversial. We evaluated the VEGF-C and vascular endothelial growth factor receptor-3 (VEGFR-3) expression in NSCLC samples from patients who had undergone surgery between 1998 and 2002 using real-time quantitative RT–PCR and immunohistochemical staining. We failed to find a positive association between VEGF-C and VEGFR-3 mRNA expression and lymph node metastasis in NSCLC. An immunohistological study demonstrated that VEGF-C was expressed not only in cancer cells, but also in macrophages in NSCLC, and that VEGFR-3 was expressed in cancer cells, macrophages, type II pneumocytes and lymph vessels. The VEGF-C/VEGFR-3 ratio of the node-positive group was significantly higher than that of the node-negative group. Immunohistochemical staining showed that VEGFR-3 was mainly expressed in cancer cells. The immunoreactivity of VEGF-C and VEGFR-3 was roughly correlated to the mRNA levels of VEGF-C and VEGFR-3 in real-time PCR. VEGF-C mRNA alone has no positive association with lymph node metastasis in NSCLC. The VEGF-C/VEGFR-3 ratio was positively associated with lymph node metastasis in NSCLC. This suggests that VEGF-C promotes lymph node metastasis while being influenced by the strength of the VEGF-C autocrine loop, and the VEGF-C/VEGFR-3 ratio can be a useful predictor of lymph node metastasis in NSCLC.

Lung cancer is the leading cause of death due to malignant disease in the developed world. The extent of lymph node metastasis is a predictor of prognosis (Mountain, 1997). Recently, the assessment of lymphangiogenesis and lymph node metastasis of neoplasms has become possible with the development of lymphatic vessel-specific markers, such as LYVE-1, Prox-1, Podoplanin and D2–40 (Banerji *et al*, 1999; Weninger *et al*, 1999; Wigle and Oliver, 1999; Kahn *et al*, 2002), and with the elucidation of the role of vascular endothelial growth factor-C (VEGF-C) and vascular endothelial growth factor receptor-3 (VEGFR-3) in lymphangiogenesis. However, many questions remain to be addressed about the mechanisms of lymphatic metastasis. VEGF-C is the lymphangiogenic factor that activates VEGFR-2 and VEGFR-3 in lymphatic endothelial cells (Jeltsch *et al*, 1997). VEGF-C/VEGFR-3 signaling plays a crucial role in the growth and survival of human lymphatic endothelial cells through mitogen-activated protein kinase and phosphatidylinositol 3-kinase signaling pathways (Makinen *et al*, 2001). Overexpression of VEGF-C in cancer cells results in the enlargement of peritumoral lymphatic vessels and in increased intratumoral lymphangiogenesis, which promotes cancer metastasis to regional lymph nodes (Skobe *et al*, 2001). A positive association of VEGF-C expression with lymph node metastasis has been reported in several cancers. However, previous immunohistochemical studies could not produce a consensus about the role of VEGF-C in non-small cell lung cancer (NSCLC) (Kajita *et al*, 2001; Arinaga *et al*, 2003; Ogawa *et al*, 2004). Therefore, we assessed the expression of VEGF-C and VEGFR-3 mRNA in NSCLC by real-time quantitative RT–PCR to demonstrate how they correlated with clinicopathological factors, including lymph node metastasis.

## MATERIALS AND METHODS

### Patients

Tissue samples were obtained from 74 patients with NSCLC who underwent surgery at Tokushima University Hospital from 1998 to 2002. Patients who underwent preoperative therapy were excluded. Tissue samples were immediately frozen in liquid nitrogen and stored at −80°C until RNA extraction. The patients included 54 males and 20 females, and they ranged in age from 33 to 83 (average age, 67 years). The pathological types were 39 adenocarcinomas, 32 squamous cell carcinomas and three large cell carcinomas, according to World Health Organization (WHO) recommendations (The World Heath Organization histological typing of lung tumors. Second edition, 1982). The pathological stages were classified as stage I in 39 patients, II in 12, III in 20 and IV in three, according to the International Staging System for Lung Cancer (Mountain, 1997). (Table 1).

### RNA extraction and cDNA synthesis

Total RNA was extracted from tissue specimens using the RNeasy Kit (Qiagen, Tokyo, Japan) according to the recommendations of the manufacturers protocol. Complementary DNA (cDNA) was transcribed from the mRNA using Sensiscript Reverse Trancriptase (Qiagen, Japan) and Oligo-dt primers (Gibco BRL, Karlsruhe, Germany) according to the manufacturer's protocols.

### Real-time quantitative PCR

The levels of VEGF-C mRNA, VEGFR-3 mRNA, and glyceraldehyde-3-phosphate dehydrogenase (GAPDH) mRNA were determined by real-time quantitative PCR. cDNA samples (2 *μ*g) were used as the template for amplification reactions carried out with the LC Fast Start DNA master Sybr Green I kit (Roche Applied Science, Mannheim, Germany) following the manufacturer's instructions. PCR amplification was carried out in a Light Cycler System (Roche, Mannheim, Germany). After an initial denaturation for 10 min at 95°C, the samples were run for 40 cycles at 95°C (15 s), 59°C (10 s) and 72°C (12 s). Data was analysed with LightCycler software 3 version 3.5.28 (Idaho Technology Inc.). For analysis purposes, the amplicon for each of the analysed genes was cloned, and known amounts of the cloned product were used to generate a standard curve. The number of copies of the gene of interest in each sample was extrapolated from the corresponding standard curve using the indicated software. For each sample, duplicate determinations were made, and mean values were adopted for further calculations. GAPDH was used as an internal control. Values of VEGF-C and VEGFR-3 mRNA were divided by that of GAPDH mRNA from the same sample. The sequences of the primers were (5′–3′): VEGF-C, forward (5′-GACTCAACAGATGGATTCC-3′) and reverse (5′-GGGCAGGTTCTTTTACAT-3′); VEGFR-3, forward (5′-CTACAAAGACCCCGACTACG-3′) and reverse (5′-CGTAGTCGGGGTCTTTGTAG-3'); and GAPDH, forward (5′-CAACAGCCTCAAGATCATCAGC-3') and reverse (5′-TTCTAGACGGCAGGTCAGGTC-3′).

We confirmed that each PCR product of VEGF-C, VEGFR-3 and GAPDH was a single band by agarose gel before Light Cycler System examination.

### Immunohistochemistry

Immunohistochemical stainings for VEGF-C and VEGFR-3 were performed using the streptavidin–biotin–peroxidase complex method. Formalin-fixed and paraffin-embedded sections of tumour tissues from resected lung were cut to a thickness of 4 *μ*m and placed on silane-coated slides. After deparaffinization in xylene and rehydration in graded ethanol, endogeneous peroxydase was blocked by incubation with 3% hydrogen peroxidase for 30 min. Tissue sections were autoclaved at 121°C in 100 mM citrate buffer, pH 6.0, for 20 min to expose the antigenic epitopes, and cooled at room temperature for 30 min. The sections were immersed in normal goat serum for 15 min at room temperature. Sections were incubated for 12 h at 4°C with a 1 : 40 dilution of anti-VEGF-C rabbit polyclonal antibody (Santa Cruz Biotechnology, Santa Cruz, CA, USA) or with a 1 : 100 dilution of anti-VEGFR-3 rabbit polyclonal antibody (Santa Cruz Biotechnology, Santa Cruz, CA, USA), followed by incubation with biotinylated secondary antibody, and streptavidin–biotin complex (DAKO LSAB+system, DAKO Co, Carpinteria, CA, USA). The sections were developed with diaminobenzidine and nuclei were counterstained with haematoxylin.

### Statistical analysis

The data were expressed as means±s.d.s. An unpaired *t*-test was used to analyse VEGF-C and VEGFR-3 mRNA expression, and the VEGF-C/VEGFR-3 ratio for each clinicopathological factor; age (70< *vs* 70⩾), gender (male *vs* female), T factor (T1 *vs* T2), N factor (N− *vs* N+), stage (I and II *vs* III and IV), histological type (adenocarcinoma *vs* squamous cell carcinoma), differentiation (well *vs* moderate and poor). A paired *t*-test was used to analyse VEGF-C and VEGFR-3 mRNA expression between 10 pairs of normal and lung cancer tissues (SPSS, version 11.0; SPSS Inc., Chicago, IL, USA). *P*<0.05 was considered statistically significant.

## RESULTS

### Expression levels of VEGF-C and VEGFR-3 mRNA in normal lung and lung cancer tissues

We examined VEGF-C and VEGFR-3 mRNA expression levels in 10 pairs of samples of tumour tissues and normal lung tissues that were distant from the primary tumour. The VEGF-C expression level was higher in the normal lung tissues than in the tumour tissues (130.2±59.7 *vs* 65.1±46.1, *P*=0.03), while there was no significant difference in the VEGFR-3 expression level between these tissues (53.5±101.4 *vs* 42.1±33.4, *P*=0.73). Relative expression levels of VEGF-C and VEGFR-3 mRNA in 74 tumour tissues were 42.9±51.7 and 52.7±99.1, respectively.

The relationship between VEGF-C or VEGFR-3 mRNA expression and clinicopathological features are shown in Table 2. The expression level of VEGF-C mRNA in adenocarcinoma was significantly higher than that in squamous cell carcinoma (*P*=0.006). For the N-factor, the node-positive group tended to have a lower VEGF-C mRNA expression level than the node-negative group (*P*=0.08). There was no significant correlation between VEGF-C mRNA and the other clinicopathological factors, including age, gender, T-factor, stage and differentiation. The node-positive group had significantly lower VEGFR-3 mRNA expression levels than the node-negative group (*P*=0.003). The expression level of VEGFR-3 mRNA in adenocarcinoma was significantly higher than that in squamous cell carcinoma (*P*=0.003). There was no significant correlation between the VEGFR-3 mRNA level and other clinicopathological factors (Table 2).

### VEGF-C and VEGFR-3 protein levels in normal lung and lung cancer tissues

**Figure 1:**
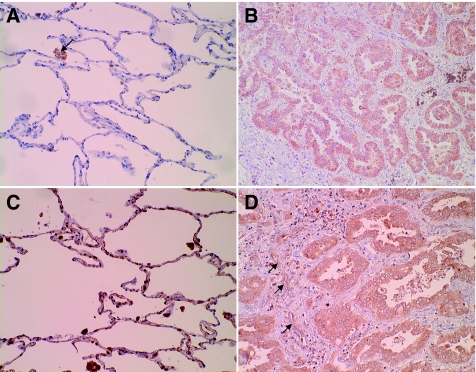
(**A**–**D**) Immunohistochemical staining for VEGF-C and VEGFR-3. (**A**) In normal lung tissue, VEGF-C was observed in macrophages (arrow). (**B**) In tumor tissue, VEGF-C was mainly observed in the cytoplasm of the cancer cells and macrophages infiltrated into the stromal tissues. (**C**) In normal lung tissue, VEGFR-3 was observed in alveolar type II cells and macrophages. (**D**) In tumour tissue, VEGFR-3 immunoreactivity was present in the cytoplasm of cancer cells, macrophages and endothelial cells of lymphatic vessels in the stroma surrounding the cancer cells (arrows).

To evaluate the location of VEGF-C and/or VEGFR-3 positive cells, we assessed the VEGF-C and VEGFR-3 protein levels in 14 pairs of lung cancer tissues and adjacent normal lung tissues by immunohistochemical methods. VEGF-C was mainly observed in the cytoplasm of cancer cells and macrophages that had infiltrated into the stromal tissues. Strong VEGFR-3 immunoreactivity was frequently present in the cytoplasm of cancer cells. VEGFR-3 immunoreactivity was also present in macrophages and endothelial cells of lymphatic vessels in the stroma surrounding the cancer cells. When we classified the cases in which more than 50% of the cancer cells were stained as positive cases, there were seven positive cases (50%) for VEGF-C and six positive cases (43%) for VEGFR-3. In normal lung tissues, VEGF-C was observed in macrophages, and VEGFR-3 was observed in alveolar type II cells and macrophages (Figure 1). The VEGF-C mRNA expression levels in the tumour tissues with (*n*=7) and without (*n*=7) VEGF-C protein expression were 52.5±53.2 and 28.8±31.0, respectively. VEGFR-3 mRNA expression levels in the tumour tissues with (*n*=6) or without (*n*=8) VEGFR-3 protein expression were 73.4±83.2 and 14.5±17.1, respectively. The immunoreactivity of VEGF-C and VEGFR-3 in cancer cells roughly correlated to the relative levels of VEGF-C and VEGFR-3 mRNA expression.

### VEGF-C/VEGFR-3 ratio of lung cancer tissues

In order to evaluate the balance of VEGF-C and VEGFR-3 mRNA expression levels, we measured the ratio of VEGF-C/VEGFR-3 mRNA expression levels in each lung cancer (Table 3). The VEGF-C/VEGFR-3 ratio in the node-positive group was significantly higher than that of the node-negative group (*P*=0.03). The VEGF-C/VEGFR-3 ratio was associated significantly with gender and histological type (*P*=0.006 and 0.04). There was no significant correlation between VEGF-C mRNA and other clinicopathological factors, including age, T-factor, stage or differentiation.

## DISCUSSION

A positive association of VEGF-C expression with lymph node metastasis has been reported in head and neck cancer, thyroid cancer, lung cancer, oesophageal cancer, pancreatic cancer, gastric cancer, colorectal cancer, ovarian cancer and prostatic cancer (Bunone *et al*, 1999; Tsurusaki *et al*, 1999; Yonemura *et al*, 1999; Kajita *et al*, 2001; Kitadai *et al*, 2001; Tang *et al*, 2001; Kawakami *et al*, 2003; Neuchrist *et al*, 2003; Yokoyama *et al*, 2003). However, some types of cancer, including lung cancer, did not demonstrate a positive association of VEGF-C with lymph node metastasis (Arinaga *et al*, 2003; van Trappen *et al*, 2003; Ogawa *et al*, 2004).

This study demonstrated that VEGF-C was expressed not only in cancer cells, but also in macrophages in NSCLC, using immunohistochemistry, and that VEGFR-3 was expressed in cancer cells and macrophages, type II pneumocytes and lymph vessels. Lung cancer cells expressed not only VEGF-C, but also VEGFR-3. Several previous studies of NSCLC demonstrated that VEGF-C is expressed in tumour cells (Kajita *et al*, 2001; Arinaga *et al*, 2003; Ogawa *et al*, 2004) and stromal macrophages (Arinaga *et al*, 2003; Ogawa *et al*, 2004), and that VEGFR-3 was observed in the cytoplasm of tumour cells (Kajita *et al*, 2001; Arinaga *et al*, 2003), lymphatic vessels (Niki *et al*, 2000; Kajita *et al*, 2001) and macrophages (Arinaga *et al*, 2003) using immunohistochemical methods. Our immunohistochemical results in NSCLC agree with these previous results. Therefore, we expected that a complex interaction was occurring between VEGF-C and VEGFR-3 in both normal and cancer cells in NSCLC. To comprehensively evaluate this interaction between VEGF-C and VEGFR-3 in the complex cellular environment, we assessed the expression levels of VEGF-C and VEGFR-3 mRNA by real-time quantitative RT–PCR in NSCLC.

Our results failed to find a positive association between VEGF-C and VEGFR-3 mRNA expression levels and lymph node metastasis in NSCLC. Patients with lymph node metastasis tended to have a lower VEGF-C mRNA expression level than those without metastasis. Arinaga and Ogawa also reported that there was no significant correlation between the VEGF-C expression level and lymph node metastasis (Arinaga *et al*, 2003; Ogawa *et al*, 2004) On the other hand, Kajita demonstrated a positive association between VEGF-C expression level and lymph node metastasis using immunohistochemical methods (Kajita *et al*, 2001). These findings suggest that VEGF-C expression levels in the tumour alone cannot predict lymph node metastasis in NSCLC. We suspect that this may be due to the complex environment of the VEGF-C and VEGFR-3 interaction in NSCLC. This study demonstrated that some lung cancers expressed both of VEGF-C and VEGFR-3 at the protein (29%: data not shown) and mRNA level (19%). This data suggests that there is an autocrine pathway between VEGF-C and VEGFR-3 in NSCLC.

Some studies demonstrated an autocrine pathway between VEGF-C and VEGFR-3 in cervical cancer and mesothelioma. Van Trappen reported that more than 50% of cervical cancer in an advanced stage, which frequently had lymphogenous metastasis, expressed VEGF-C and/or VEGFR-3 proteins, and that this suggested an autocrine growth stimulation pattern via VEGFR-3 in cervical cancer (van Trappen *et al*, 2003). Masood *et al* (2003) reported that reduced levels of VEGF-C inhibit mesothelioma cell growth *in vitro*, and that antibodies to VEGFR-3 inhibited mesothelioma cell growth. These results indicated that a functional VEGF-C autocrine growth loop is functioning in mesothelioma cells. We speculate that in tumour cells that express both VEGF-C and VEGFR-3, the secreted VEGF-C mainly binds to VEGFR-3 on the tumour cells themselves (autocrine loop), and that only residual VEGF-C binds to the VEGFR-3 receptors in lymphatic endothelial cells (paracrine pathway) and promotes lymphangiogenesis. Schoppmann *et al* (2002) reported that stromal macrophages expressed VEGF-C and played important roles in peritumoral lymphangiogenesis. We suspect that the balance of VEGF-C and VEGFR-3 expression in the environment of NSCLC affects the lymphangiogenesis. When the amount of VEGF-C secreted by the tumour cells and macrophages overtakes the amount of VEGFR-3 in cancer cells, macrophages and type II pneumocytes, lymphangiogenesis is promoted and lymphogenous metastasis is increased. In this study, we evaluated the relative amount of VEGF-C and VEGFR-3 expression in tumour tissues and lymphogenous metastasis in NSCLC. The VEGF-C/VEGFR-3 ratio of the node-positive group was significantly higher than that of the node-negative group (3.66±4.05 *vs* 1.77±2.44, *P*=0.03). The VEGF-C/VEGFR-3 ratio is a parameter of the balance between VEGF-C and VEGFR-3 expression in tumour tissues. This result suggests that when VEGF-C expression is relatively higher than VEGFR-3 expression, lymph node metastasis is promoted. A previous report demonstrated that the serum VEGF-C level of patients with lymph node metastasis was significantly higher than that of patients without metastasis in NSCLC (Tamura and Ohta, 2003). This study supports our speculation. We think that when the serum VEGF-C level is excessive, and excess VEGF-C cannot bind to VEGFR-3 in the cancer cells, it binds to VEGFR-3 in lymph vessels, promoting lymphangiogenesis and lymph node metastasis.

In conclusion, tumour cells expressed both VEGF-C and VEGFR-3 in NSCLC (autocrine loop), and other normal cells (macrophages, type II pneumocytes and lymph vessels) also expressed VEGF-C and/or VEGFR-3. In this complex environment, VEGF-C expression levels alone cannot predict lymph node metastasis. The balance of VEGF-C and VEGFR-3 expression levels in the tumour tissues affects the lymph node metastasis. The VEGF-C/VEGFR-3 ratio can be a useful predictor of lymph node metastasis in NSCLC.

## Figures and Tables

**Table 1:** Patient characteristics

**Characteristic**	**No. of patients**
Age (mean±s.d.)	67±10
*Gender*	
Male	54
Female	20
	
*TNM*	
*T*	
1	28
2	32
3	5
4	9
*N*	
0	48
1	12
2	14
*M*	
0	71
1	3
	
*Stage*	
I	39
II	12
III	20
IV	3
	
*Histological type*	
Adenocarcinoma	39
Squamous cell ca	32
Large cell ca	3
	
*Differentiation*	
*Adenocarcinoma*	
Well	16
Moderate	17
Poor	6
*Squamous cell ca*	
Well	6
Moderate	19
Poor	7

**Table 2:** Correlation between VEGF-C and VEGFR-3 mRNA expression and clinicopathological factor

**Clinicopathological factors**	**No. of patients**	**VEGF-C**	***P*-value**	**VEGFR-3**	***P*-value**
*Age*					
70>	40	50.8±58.3	0.15	70.8±128.2	0.07
70⩽	34	33.6±41.5		31.3±38.0	
Gender					
Male	54	41.2±49.1	0.64	35.4±53.4	0.1
Female	20	47.5±59.3		99.3±163.3	
					
*T*					
1	28	51.7±62.3	0.17	85.9±148.9	0.09
2	32	32.5±44.8		34.4±43.7	
					
N					
(−)	48	50.6±57.0	0.08	71.7±118.2	0.003
(+)	26	28.7±37.0		17.5±21.6	
					
*Stage*					
I, II	51	48.3±57.5	0.11	66.1±116.1	0.08
III, IV	23	31.0±33.6		22.9±25.0	
					
*Histological type*					
Adenocarcinoma	39	56.6±60.4	0.006	81.3±126.8	0.003
Squamous cell ca	32	24.5±33.1		15.3±24.4	
					
*Differentiation*					
Adenocarcinoma					
Well	16	53.0±55.3	0.76	120.2±177.3	0.11
Mod or Poor	23	59.1±64.8		35.9±54.1	
*Squamous cell ca*					
Well	6	26.4±38.7	0.88	18.3±19.5	0.75
Mod or Poor	26	24.0±32.6		14.7±25.7	

VEGF-C: vascular endothelial growth factor-C;

VEGFR-3: vascular endothelial growth factor receptor-3.

**Table 3:** Correlation between VEGF-C/VEGFR-3 and clinicopathological factors

**Clinicopathological factors**	**No. of patients**	**VEGF-C/VEGFR-3**	***P*-value**
*Age*			
70>	40	2.24±2.89	0.58
70⩽	34	2.66±3.57	
			
*Gender*			
Male	54	2.88±3.57	0.006
Female	20	1.24±1.38	
			
*T*			
1	28	1.79±2.22	0.27
2	32	2.72±3.84	
			
*N*			
(−)	48	1.77±2.44	0.03
(+)	26	3.66±4.05	
			
*Stage*			
I, II	51	2.19±2.84	0.34
III, IV	23	2.97±3.91	
			
*Histological type*			
Adenocarcinoma	39	1.70±2.02	0.04
Squamous cell ca	32	3.40±3.40	
			
*Differentiation*			
Adenocarcinoma			
Well	16	1.08±1.12	0.08
Mod or Poor	23	2.12±2.39	
Squamous cell ca			
Well	6	1.58±0.92	0.24
Mod or Poor	26	3.82±4.49	

VEGF-C: vascular endothelial growth factor-C;

VEGFR-3: vascular endothelial growth factor receptor-3.
